# Efficacy of a novel sensory discrimination training device for the management of phantom limb pain: protocol for a randomised placebo-controlled trial

**DOI:** 10.1136/bmjopen-2025-101657

**Published:** 2025-11-09

**Authors:** Sarah Oatway, Denis Martin, Andrew Graham, Alan Batterham, Alasdair MacSween, Sally Smith, Deepak Ravindran, Cormac Ryan

**Affiliations:** 1 Centre for Rehabilitation, Lifestyle Medicine and Human Performance, Teesside University, Middlesbrough, UK; 2 Teesside University SSSHL, Middlesbrough, UK; 3 James Cook University Hospital, Middlesbrough, UK; 4 Royal Berkshire NHS Foundation Trust, Reading, UK

**Keywords:** Amputation, Surgical, Pain management, Chronic Pain, Clinical Trial, Neurological pain, Self-Help Devices

## Abstract

**Introduction:**

Many amputees experience phantom limb pain (PLP). Pharmacological management is the mainstay of treatment, but effectiveness is limited, and it is associated with significant side effects. Sensory discrimination training (SDT) is a non-pharmacological treatment for PLP. Previously, SDT required a clinician, or carer, to administer it, creating a barrier to real world use. In this trial, an automated SDT device (SP1X, 2pd Ltd, Middlesbrough, United Kingdom) for the self-management of PLP will be investigated for efficacy.

**Methods and analysis:**

The Phantom Relief is a decentralised, randomised, placebo-controlled, mixed-methods, superiority trial. Participants will take part from their own homes, using an electronic data capture tool to complete all trial documentation. Eligible, consenting individuals with PLP (intensity rated as ≥4 on a 0–10 scale; n=100) will be randomised to receive the SP1X device (intervention group) or a placebo device SP1X7 (placebo group). The first and second treatment sessions will be observed via video call to provide set-up guidance and any additional advice needed. The primary outcome measure will be the McGill Pain Questionnaire revised (SF-MPQ-2). Outcome measures will be collected at baseline, 3 weeks (immediately post intervention) and 3 months follow-up. Statistical analysis will be carried out by a blinded statistician (analysis of covariance model conditioning on the baseline and stratification factors). Semi-structured interviews will be carried out with a sub-sample (n=10–15) of intervention group participants. Participants will be provided with their allocated device for home use. Online video calls will be used to instruct participants on how to set up and use the device by the research assistant (RA). The RA will observe the first and second treatment sessions and provide any additional advice needed. Participants in both groups will be asked to use the device for 60 min/day for at least 15 days of the 21-day treatment period and to record device use in a study diary.

**Ethics and dissemination:**

Approval has been obtained from Teesside University School of Health and Life Sciences Research and Ethics Committee, the North of Scotland Research Ethics Service, Health Research Authority, and a letter of no objection was obtained from the Medicines and Healthcare products Regulatory Authority. The results will be disseminated through peer-reviewed articles, conference presentations and a doctoral thesis.

**Trial registration number:**

NCT04103983.

**STRENGTHS AND LIMITATIONS OF THIS STUDY**

- This trial will involve an appropriate number of participants (n=100) to provide adequate power for detecting a moderate clinically relevant effect size.
- The trial will recruit UK-wide and both upper and lower limb amputees are eligible, enhancing generalisability.
- As a decentralised trial, participants will self-apply the device in their own homes, mirroring the intended real-world use.
- Necessity for access to smart devices may increase digital exclusion from participation.
- Due to the requirement to instruct on device use, the research assistant is not blind to group allocation post randomisation.

## Introduction

 Phantom limb pain (PLP) is defined as any noxious sensory phenomenon in the missing body part that develops after surgical amputation of a limb.^1 2^ PLP is a long-term condition that affects 76%–87% of amputees.^3^ The pain is persistent in nature and can impair activities of daily living, quality of life and ability to work.^4^,^6^ Pharmacological management is the mainstay of current treatment; however, evidence of effectiveness remains inconclusive and many people experience significant side effects.^7^ Those include: dizziness, tiredness, constipation, sweating, nausea, vertigo, sedation, itching and shortness of breath.^8 9^ Due to the ambiguity of evidence, costs (especially when used over the long term) and the high occurrence of side effects, a greater focus on non-pharmacological conservative therapies is warranted. However, there is also limited evidence on the effectiveness of non-pharmacological interventions, and the evidence that does exist is of low quality.^10^,^12^

The underlying pathophysiology of PLP is not fully understood, although it is hypothesised to arise from a combination of peripheral, central and cortical mechanisms. One of the mechanisms proposed to underpin PLP is cortical (re)organisation following amputation.^13^ Multiple studies^13^,^16^ have shown that after amputation, the sensorimotor cortical representations of neighbouring body areas can shift. This is known as cortical re-organisation (or maladaptive plasticity) and is one of the key neural mechanisms proposed to underlie PLP.^17 18^ Crucially, the extent of this shift in representation is correlated with PLP intensity.^19 20^

It was initially postulated that this was evidence of the amputated limb representation becoming diminished and being encroached by the cortical representation of neighbouring bodily areas.^13 17^ More recently, alternative brain scanning techniques have shown that the cortical representation of the amputated limb remains intact and the more intact it remains, the greater the PLP (r=0.49, p<0.01).^19 20^ This mechanism is known as the maintained representation model.^21^ Using transcranial direct current stimulation to disrupt this phantom hand representation can reduce the experience of PLP.^20^ These findings were initially thought to be contrary to the cortical reorganisation model; however, as stated by Kikkert *et al,*^22^ “It is conceptually and empirically possible that persistent representation can spatially coincide with reorganisation.” In a recent narrative review, co-authored by the leading proponents of these two models, both models are discussed as potential neural mechanisms of PLP and suggest they may be ‘complementary’ processes within the cortical (re)organisation model.^20^

Sensory discrimination training (SDT) is a non-pharmacological intervention which aims to target cortical (re)organisation. SDT involves stimulating a number of spatially close, but distinct, areas on the residual limb. Usually, this is provided manually. Previous studies have used electrical stimulation to provide this stimulus,^17^ whereas others have used vibrotactile type stimulation.^23 24^ Currently, there is no suggestion that one type of technology is superior to the other. The patient must make a judgement about some aspect of the stimulation, such as the stimulation location, and they then receive feedback informing them if that judgement was correct/incorrect.^25^ It is theorised that concentrating on stimuli from the limb alters the cortical representation, via neuroplasticity, improving the match between the physical and neural limb.

In a seminal paper, Flor *et al*^13^ reported that after a course of SDT, PLP was significantly reduced, and the magnitude of the reduction was directly correlated (r=0.73, p<0.05) to a normalising shift of the represion of a neighbouring bodily area.^17^ This finding indicates that SDT may be a beneficial treatment for PLP through cortical (re)organisation mechanisms. These initial findings are further supported by small (n≤10) studies.^26 27^ However, the quality of the literature for SDT is poor and there is a need to investigate its effectiveness using robust, appropriately powered randomised controlled trials (RCTs).^25^

To date, SDT in practice has been delivered in a manner that requires a clinician, or carer, to administer the intervention. That, coupled with the high number of treatments needed over a prolonged period (ranging from 30 min/day to 90 min/day over 2–4 weeks), makes implementation of SDT into clinical practice resource intensive, creating a considerable barrier to real world use.^10^ To overcome this practical challenge, the SP1X has been developed. The SP1X is a fully automated, interactive SDT device which is self-administered by the patient. This technology uses electrical stimulation to deliver the stimulation. To date, there have been no studies investigating the efficacy of this device.

The primary aim of this trial will be to investigate the efficacy of the SP1X for the management of PLP.

## Methods and analysis

### Study design and setting

The Phantom Relief trial is a randomised, placebo-controlled, mixed-methods, superiority trial. The trial is fully decentralised. Participants will take part from their own homes and all communications will be via email/SMS messages, telephone or video conferencing platforms. An electronic data capture (EDC) tool (Greenlight Guru, Indianapolis, Indiana 46225, US) will be used to complete all trial documentation. The start date for this study was 2 September 2024, with a completion date of 18 July 2025.

### Study population

We aim to recruit 100 people with PLP following an above ankle/wrist amputation. To be eligible for this trial, participants must meet the following key inclusion criteria: UK adults, ≥18 years of age, with a fully healed residual limb, experience of PLP intensity rated as ≥4 on a 0–10 scale on at least 2 days in the week prior to enrolment, access to a mobile smart phone and another electronic device (laptop, iPad etc). Individuals will be ineligible if they meet any of the exclusion criteria, for example: impaired sensation on the area of residual limb where the device is to be applied, active deep vein thrombosis, thrombophlebitis or varicose veins, any actively bleeding tissue or untreated haemorrhagic disorders, pregnant women, any current or recent history of substance misuse, alcohol or drug dependency, a metal implant in the area to be stimulated, unable to speak English or lack of capacity to give informed consent (online supplemental appendix 4). A full list of the inclusion and exclusion criteria is given in online supplemental appendix 1.

Participants will be recruited via a range of strategies, including postal and face-to-face clinician contact, invitations from General Practitioner practices and specialist amputee rehabilitation centres in the UK, and web-based social media and web page activity, to include e-mails and adverts, targeting relevant patient groups and charities, as well as existing mailing lists of people with PLP. Full details of the recruitment strategy are given in online supplemental appendix 2. Participant recruitment, by all routes, will use the documentation, process and sequence model advocated by Oxford ‘A’ REC (a National Health Service (NHS) Research Ethics Committee based in the UK) and all participants must provide written informed consent.

### Patient and public involvement and engagement

A trial-specific patient and public involvement and engagement (PPIE) group has been engaged and actively involved in all aspects of design and will continue to be actively involved in delivery, analysis and dissemination. This group includes service users, patient, and public expert consultees. The trial steering committee (TSC; who will advise and oversee all aspects of the trial) includes members of the PPIE group. Advice was also sought from an equality, diversity and inclusion advisor. The PPIE group reviewed and supported the final participant-facing documentation.

### Sample size

We regard the maximum feasible sample size within the constraints of this trial as a total of 100 participants. With an estimated loss to follow-up at 3 weeks (post intervention) of 20%, the effective sample size is 80 participants (40 in each group in a 1:1 allocation ratio). Our targeted effect size is a difference in post-intervention (3 week timepoint) McGill Pain Questionnaire revised (SF-MPQ-2) total score between arms of 1 unit—a minimally clinically relevant effect size. An Minimal Clinically Important Difference (MCID) for pain for people with PLP has not been definitively established. However, for chronic pain in general, according to the Initiative on Methods, Measurement, and Pain Assessment in Clinical Trials (IMMPACT) recommendations, a reduction in pain of 10%–20% can be considered to represent a ‘minimally important’ clinical change.^28^ This is in concordance with recent National Institute for Health and Care Excellence guidelines for chronic pain which have identified ‘the use of 10% of a scale’ as one appropriate option for between group differences in pain.^29^ The between-subjects variability (SD) was derived from a small reliability. Study with 37 participants drawn from the same population as the proposed trial (unpublished observations).^25^ The observed SD was 2.13 units. The pre–post correlation (with 1 week between test and retest) was 0.82. The pre–post correlation (the reliability of the measure) is expected to decrease over time. Estimates for the decline in correlation with more distant pairs of timepoints post-randomisation range from −0.003/month^30^ to −0.009/month.^31^ We have assumed a conservative value for this correlation of r=0.7 at the 3-week primary endpoint. An effective sample size of 40 participants per arm gives c.84% power at two-sided p=0.05 to detect the targeted effect size. This sample size estimate was derived assuming an analysis of covariance (ANCOVA) model, adjusting for the baseline value of the outcome measure. To allow reproducibility of our sample size estimate, the relevant Stata code is provided herein (StataCorp, 2023. Stata Statistical Software: Release 18, College Station, TX, USA: StataCorp LP): sampsi 5 4, sd1(2.13) sd2(2.13) method(ancova) pre(1) post(1) r01(0.7) n(40) n2(40).

### Randomisation

The research assistant (RA) will use the Sealed Envelope (London, United Kingdom) remote randomisation software system to randomise participants (1:1) to one of two trial groups: intervention group (SP1X) and placebo group (SP1X7). A unique participant identification code (eg, 001, 002) will be allocated to all participants. The allocation will be stratified by sex (male/ female) and chronicity (<6 months/≥6 months post surgery), generating four separate strata. Within these strata, participants will be allocated using a mixed (unrestricted and restricted) randomisation approach. This approach uses an initial simple (unrestricted) randomisation to provide unpredictability and guard against guessing,^32^ followed by allocation using randomly permuted blocks to achieve reasonable balance, resulting in approximately 50 participants each arm overall. More explicit details of the randomisation scheme are not provided herein, to avoid the facilitation of the deciphering of the allocation sequence.

### Intervention

**Figure 1:**
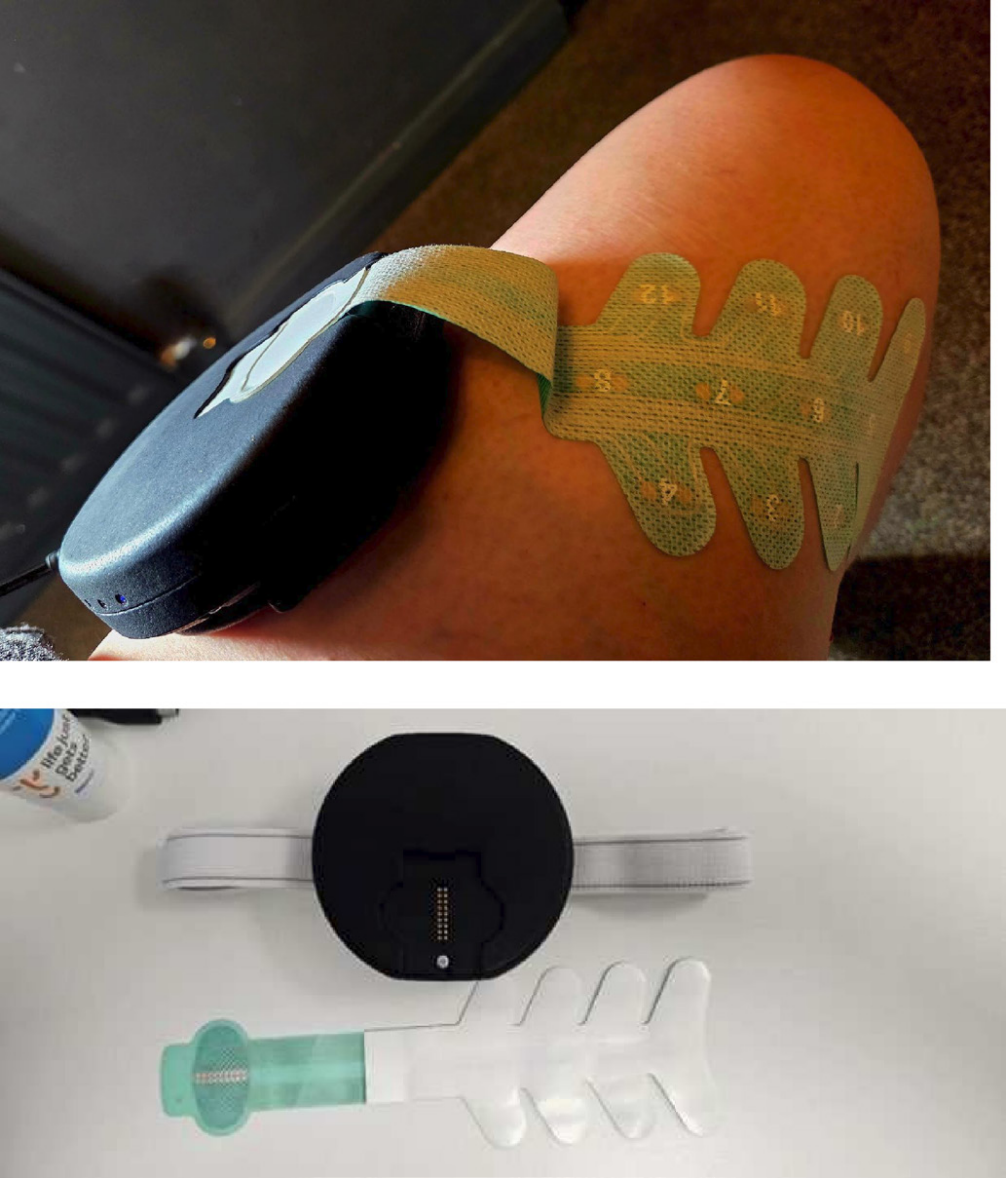
Photograph of the device.

There will be two parallel groups, an intervention group (SP1X) and a placebo group (SP1X7). Both the intervention and placebo device, which are identical in appearance, will be provided to participants for use in their own homes. A picture of the device is provided in figure 1.

During an online video call, the participant will be instructed on how to set up and use the device by the RA (a Health and Care Professions Council registered physiotherapist). Both the devices have an electrode patch which is placed on the residual limb. This pad is connected, via Bluetooth, to a separate tablet device running an Android 10+ app designed to deliver either an interactive SDT programme or a non-interactive placebo programme. The RA will observe the first full treatment session (60 min) and up to 30 min of the second treatment, and provide any additional advice needed. Participants in both groups will be asked to use the device for 60 min/day, as one block, or as multiple shorter sessions of ≥20 min duration on at least 15 days of the 21-day treatment period. Participants will receive support phone calls alongside daily texts to aid adherence to the protocol. They will be asked to spread out the use of the device over the treatment period and to record their device use in a study diary. A complete description of the intervention in alignment with the Template for Intervention Description and Replication guidelines^33^ is provided in online supplemental appendix 7.

### Primary and secondary outcome measures

#### Primary outcome

The primary outcome will be the total score of the (SF-MPQ-2)^34 35^ at the 3-week time point. The SF-MPQ-2 is a commonly used questionnaire to assess pain levels in a range of pain conditions. There are 22 items/ pain descriptors across four pain sub-scales/domains: continuous, intermittent, neuropathic and affective. Participants rate each item on an 11-point (0–10) scale, where 0=none and 10=worst possible pain. The mean of the 22 items provides the SF-MPQ-2 total score. Two or more missing responses on any sub-scale result in an invalid outcome. A targeted effect size of 1 unit difference between groups will be used.

#### Secondary outcomes

The following secondary outcomes are specified at the 3-week timepoint (immediately post intervention).

- Overall pain intensity score: visual analogue scale (100 mm).^34^
- Frequency-adjusted pain score: (0–100).
- General subjective outcome score (GSOS).^36^

#### Exploratory outcomes

No inferences will be drawn from these outcomes.

- SF-MPQ-2 total score at the 3-month time point.
- Trinity Amputation and Prothesis Experience Scales (TAPES), (modified).^37 38^
- Quality of life EuroQol 5-Dimension 5-Level (EQ-5D-5L).^39 40^
- Sleep disturbance (the Patient-Reported Outcomes Measurement Information System (PROMIS) Short Form V.1.0–Sleep Disturbance 4a questionnaire).^41^
- Participant satisfaction.^42^
- Device usability (an adapted version of the 10-question System Usability Scale (SUS)).^43^
- Study diary of device and medication usage.
- Concordance with protocol.
- Success of blinding.

Supplementary detail for these secondary and exploratory outcome measures is provided in online supplemental appendix 5.

### Blinding strategy

This design optimises the methodological quality of the trial with regards to random allocation and concealment, outcome assessor blinding and participant blinding. To facilitate participant blinding, participants will be told that there are two different devices being investigated, an interactive device and a non-interactive device. They will be told that this trial will compare both devices, as well as comparing each individual device to a placebo.

Thus, participants will be told they could be in one of four groups: (1) interactive device group, (2) interactive device placebo group, (3) non-interactive device group and (4) non-interactive placebo device group. However, in reality, there will only be an intervention group (which involves a perceptible stimulation and requires interaction with the device) and a placebo group (which involves no stimulation and no interaction with the device). In addition, participants will be informed that different electrical currents are used, including microcurrents, which are not always perceptible. We have used this approach previously in transcutaneous electrical nerve stimulation studies to blind participants.^44^ However, all participants will be aware that there is 50% chance that they will receive a placebo device, which reflects the real ratio. This deception minimises the risk that participants will identify that they have been assigned to a placebo group and has received ethics approval.

All baseline outcome measures will be completed prior to randomisation. The post-intervention and 3-month follow-up outcome data collection will be undertaken independently by the participants themselves via online forms sent automatically via the EDC platform. All the quantitative outcome measures are participant self-reported outcome measures. According to the Cochrane collaboration, where outcome measures are self-reported and have been collected under participant-blinded conditions, and the participant blinding has been confirmed to be successful, the trial can be regarded to have assessed outcomes blindly.^45^ In this trial, participants will be blinded, and the success of that blinding will be tested. Additionally, the person undertaking the statistical analysis will be blind to group allocation.

### Statistical analysis

The primary outcome data (SF-MPQ-2 total score) will be analysed using an ANCOVA model, conditioning on the baseline value of the outcome, in a linear mixed model with restricted maximum likelihood and Satterthwaite df. This model is one of the principled approaches to dealing with missing outcome data under a missing at random assumption; provided a participant has outcome data for at least one of the two timepoints, then they will be included in the analysis. Given the nature of this trial, we do not anticipate missing baseline data but will apply a suitable approach if so.^46^ Fixed effects will be time (3 weeks and 3 months), treatment group, and the time-by-treatment interaction, adjusting for baseline SF-MPQ-2. Alongside the fixed effect for baseline, we shall include a baseline-by-time interaction to allow for a different association between baseline and outcome at each timepoint. To preserve the desired type 1 error control, an indicator variable (fixed factor) for membership of the four strata defined by the two, two-level stratification factors will be included in the model.^47^ We specify the most general and flexible ‘unstructured’ residual correlation matrix accounting for the repeated measurements nested within participant, imposing no constraints and estimating a variance for each timepoint and their covariance. Therefore, the random participant intercept is omitted (‘noconstant’). The primary endpoint will be the estimate of the effect of the intervention at 3 weeks. We shall present the point estimate together with 95% CI and p value, via t-based inferences using Satterthwaite df. The CI provides a plausible range of effect sizes compatible with the data, model and underlying statistical assumptions and represents an estimation approach. All analyses will be conducted using Stata software (Release 18). Sample draft Stata code for this primary analysis is provided below: mixed sfmpq2 i.group##i.time c.baseline##i.time i.indicator||id:, noconstant residuals(unstructured, t(time)) reml dfmethod(satterthwaite)

The behaviour of the model residuals will be inspected visually using residuals plots, with data transformations applied if indicated. An intention-to-treat analysis (primary) and a per-protocol analysis (secondary) will be undertaken. In the per-protocol analysis, the participant will be required to have undertaken at least 10 sessions of SDT totalling (600 min) to be considered suitable for inclusion in the analysis. The primary analysis will be carried out by a statistician blinded to group assignment.

The three secondary outcome measures will be treated as continuous and analysed in the same way, but obviously with no baseline value for the GSOS. The secondary outcomes are considered equally important, after a significant primary. Type 1 error for the analysis of the secondary outcomes will be preserved using the Holm multiple testing algorithm.^48^,^50^ Study diary data will be summarised using descriptive statistics.

Secondary analysis: treatment heterogeneity will be explored in a secondary analysis (non-blinded). By inclusion of appropriate interactions with treatment group, we will explore putative effect modifiers, including baseline value of outcome, age, sex and total treatment time (min) with the device. The trial is not powered for quantifying treatment heterogeneity, so we emphasise that this secondary analysis will be purely exploratory/descriptive.

Qualitative analysis: a purposive sample of participants from the active intervention group will be invited to undergo a semi-structured interview. During these interviews, the participants will be asked about the usability and acceptability of the SP1X device. The interviews will be audio-recorded, transcribed verbatim and analysed thematically. Thematic analysis will be undertaken by the RA using the method of Braun and Clarke.^51^ The topic prompts for the semi-structured interviews are provided in online supplemental appendix 8.

### Methodological limitations

Although group allocation is unknown at the point of baseline data collection, the RA will be unblinded to group allocation post randomisation. This unblinding is unavoidable, as the participants need to be provided with their specific device and receive instruction and guidance on how to use it, and the instructions needed for each of the two devices are different. The RA will provide feedback and information to participants in a neutral way and focus on correct usage of the device, giving no indication of group allocation. The RA will not be involved in the post-intervention or 3-month follow-up point outcome data collection; that will be undertaken by the participants themselves via the EDC platform.

## Ethics and dissemination

### Ethical considerations

The main ethical consideration of this trial is the degree of deception required to use a participant-blinded RCT model. When entering the trial, participants will be informed that the trial is comparing two different versions of a new treatment device—an interactive device and a non-interactive device—to facilitate blinding. Once all participants have completed the 3-month follow-up outcome measure time point, the trial participants will be debriefed, face-to-face, over a video call, informing them of the deception and which device they received. This process was discussed and agreed with our PPIE group.

This trial was registered on ClinicalTrials.gov prior to commencement. To minimise the risk of participants reading the protocol during their participation and unmasking the blinding process prematurely, the registered protocol will be in accordance with the participant information sheet and informed consent forms (ICFs) ethically approved. The full unblinded protocol will be uploaded once the trial is complete. This is based on advice from ClinicalTrials.gov. In addition, the trial protocol will be registered on the Health Research Authority (HRA) website.

This form of intervention (self-administered and/or automated SDT) is not routinely provided within the NHS and there is no robust evidence on efficacy. Furthermore, there are no studies that have investigated the efficacy of the SP1X device. Adequate equipoise, with indications of possible benefit, exists to warrant an RCT.

### Quality control and quality assurance procedures

The chief investigator (CI) and RA will be primarily responsible for quality control and quality assurance. As part of the fidelity checking process, the CI will meet with all participants to observe them using the device to ensure it is being used according to instruction. Formal monitoring of the trial will be undertaken by an independent data monitoring (and ethics) committee and the TSC. Details of the operating procedure and roles within the committees are outlined in online supplemental appendix 3.

The representative of the sponsor will have oversight of the conduct of the CI and investigators. The finance department of the sponsor will oversee financial propriety and administer all trial funds.

### Reporting of adverse events

All discovered/ disclosed adverse events (AEs), and adverse device events (ADEs), serious AEs (SAEs), serious ADEs (SADEs) and unexpected SADEs (USADEs) will be recorded by the RA on the EDC platform, which follows ISO14155:2020 AE and MEDDEV 2.7/3 reporting processes. AEs and ADEs will be reported to the CI and the sponsor’s representative by automatic notification from the EDC. The CI will send the Chair of the DM(E)C all AE and ADEs not later than within 2 working days of discovery/disclosure. All SAEs, SADEs and USADEs will be recorded immediately but preferably not later than within 2 calendar days of discovery/disclosure on the EDC platform. Non-person identifiable summary statistics for SAEs, SADEs and USADEs may be included in disseminations and/or further regulatory/licensing applications. A full description of AE reporting can be found in online supplemental appendix 6.

### Data handling and record keeping

An encrypted electronic copy of the link (coding list) document will be held on a password-protected allocated section of a Teesside University (TU) server. The electronic copies of the signed ICFs (e-ICFs), AE and contact consent forms will be held on a password-protected folder on the section of the TU server allocated to the CI. The pseudonymised trial data will be held on the personal password-protected section of a TU server allocated to the CI, and will be securely and permanently deleted from the EDC platform once complete.

During the trial, all data collected on the EDC platform will be linked to the identifiable details of the participants and thus will be person identifiable. However, the EDC will only be accessible by the CI and the RA (as well as those with an oversight audit role). On completion of the trial, the data will be rendered pseudonymised and retained on a TU password-protected server. The e-ICFs, all safety data records, contact forms and coding document will be held separately from trial data. All non-person identifiable data will be archived and made publicly available in the TU Research Data Repository (https://researchdata.tees.ac.uk/research-data/) in accordance with the TU Research Data Management Policy.

### Ethics approval and consent to participate

Documented approval has been obtained from Teesside University School of Health and Life Sciences Research and Ethics Committee (16692), the North of Scotland Research Ethics Service, HRA (23/NS/0085 IRAS 250337), and letter of no objection obtained from the Medicines and Healthcare products Regulatory Authority (IRAS 2 50 337 CI/2023/0055 /GB).

### Dissemination plan

The protocol publication is part of a variety of methods that will be carried out to achieve maximum visibility of our work. Participants will be informed in the participant information sheet that they will be sent a summary of the results and invited to a local dissemination event where the findings will be presented. The sponsor is fully committed to the dissemination of all results of all studies it conducts regardless of findings. A doctoral thesis will be prepared on this subject. The device manufacturers have funded the trial and have no right to veto or amend any aspect of analysis or interpretation of results or any dissemination.

## Supplementary material

10.1136/bmjopen-2025-101657 online supplemental file 1

10.1136/bmjopen-2025-101657 online supplemental file 2

10.1136/bmjopen-2025-101657 online supplemental file 3

10.1136/bmjopen-2025-101657 online supplemental file 4

10.1136/bmjopen-2025-101657 online supplemental file 5

10.1136/bmjopen-2025-101657 online supplemental file 6

10.1136/bmjopen-2025-101657 online supplemental file 7

10.1136/bmjopen-2025-101657 online supplemental file 8
