# Supplementary material for: Efficacy of a novel sensory discrimination training device for the management of phantom limb pain: protocol for a randomised placebo-controlled trial
Source: BMJ Open. 2025 Nov 9;15(11):e101657. doi: 10.1136/bmjopen-2025-101657 (PMC12598989; doi:10.1136/bmjopen-2025-101657)
Supplement: online supplemental file 1 [file bmjopen-15-11-s001.docx]

**APPENDIX 1**

**Inclusion and Exclusions Criteria**

Inclusion criteria

- Living in the UK
- All Genders
- Aged ≥18 years of age
- Women who self-declare:
  - that they are post-menopausal, or permanently sterile*,

- or -

- - that it is physiologically impossible that they could be pregnant and not be aware they are.
    - Women who *do not* make one of the two declarations above, are only eligible if:
      - they undertake a highly sensitive urine pregnancy test which is negative, prior (and as close in time as is possible) to beginning the treatment phase of the Trial,

- and –

- - - - they agree to use a highly, or acceptably effective, contraceptive measure** during the treatment phase of the Trial.
- fully healed residual limb (or stump) ***
- single or multi limb amputation with the intervention applied to the participants limb of choice
- experienced PLP rated as ≥4 on a 0-10 scale on at least 2 days in the week prior to enrolment
- agree to inform us of the use of any new (to them) prescribed drug for their pain during the trial
- any prescribed pharmacological treatment for the treatment of PLP stable for one month prior to commencing the trial , and agree not to undertake any non-pharmacological treatments for their PLP during the trial (e.g., mirror therapy)
- agree to inform us of any other health care received related to the amputated limb during the trial (e.g., physiotherapy or occupational therapy) whether specific to PLP or not
- any previous non-pharmacological PLP treatment must have terminated at least 1 month prior to commencing the trial
- Participants will need access to a mobile smart phone and a device (laptop, iPad etc.) at home to receive text messages and take part in secure video conference calls and completion of online forms.

*Post menopause is defined as no menses for 12 months without an alternative medical cause. Permanently sterile methods include hysterectomy, bilateral salpingectomy, and bilateral oophorectomy^22^

**Highly effective contraceptive methods being:

- - - Combined (estrogen and progestogen containing) hormonal contraception associated with inhibition of ovulation either oral, intravaginal, or transdermal
    - Progestogen only hormonal contraception associated with inhibition of ovulation, either oral, injectable, or implantable
    - Intrauterine device IUD
    - Intrauterine hormone-releasing system IUS
    - Sexual abstinence

Acceptably effective contraceptive methods being:

- - - Progestogen-only oral hormonal contraception, where inhibition of ovulation is not the primary mode of action
    - Male or female condom with or without spermicide
    - Cap, diaphragm or sponge with spermicide
    - A combination of male condom with either cap, diaphragm or sponge with spermicide (double barrier methods)^27^

******* Fully healed criteria: no bleeding, no oozing, no broken skin, no obvious sign of infection, such as swelling or redness, around the scar.

**Exclusion criteria**

- lacking Mental Capacity to give Informed Consent
- women who self-declare that they are pregnant, or that they will be trying to become pregnant, during the treatment phase of the Trial
- impaired sensation as measured by hot/ cold test and sharp/ blunt test
- unable to read and speak English - the questionnaires being used have not been translated and validated in multiple languages and no facility is available to conduct nor translate/ back-translate semi-structured interview data
- epileptic
- active deep vein thrombosis, thrombophlebitis, or varicose veins
- fitted with a pacemaker
- has a metal implant in the area to be stimulated
- any residual limb complications such as cellulitis, wounds, infections etc.
- Active regions of known or suspected malignancy
- Any actively bleeding tissue or to persons with untreated haemorrhagic disorders
- Participating in any research trial of any intervention hypothesised to affect PLP
- Any current or recent history of substance misuse, alcohol, or drug dependency
- Any person, otherwise eligible, who commences any non-pharmacological treatment for PLP during the trial period
