# Supplementary material for: Efficacy of a novel sensory discrimination training device for the management of phantom limb pain: protocol for a randomised placebo-controlled trial
Source: BMJ Open. 2025 Nov 9;15(11):e101657. doi: 10.1136/bmjopen-2025-101657 (PMC12598989; doi:10.1136/bmjopen-2025-101657)
Supplement: online supplemental file 2 [file bmjopen-15-11-s002.docx]

**APPENDIX 2**

**Recruitment Strategy**

The GP practices (via the Clinical Research Network, who will identify and approach appropriate GP Practices once research ethics and governance approvals have been gained) will send recruitment packs (Invitation letter, Key Facts Summary Sheet, Participant Information Sheet, and a copy of the Consent Form) to eligible individuals identified from their records. A reminder letter will be sent two weeks later.

PACE rehabilitation will send recruitment packs to eligible individuals identified from their records. A reminder letter will be sent two weeks later.

Clinicians working in clinics for people who have had an amputation at James Cook University Hospital, Middlesbrough, The Freeman Hospital, Newcastle, University Hospital Hartlepool, Hartlepool, and Seacroft Hospital, Leeds, will either provide an invitation letter Key Facts Summary Sheet, Participant Information Sheet, and a copy of the consent form, or gain consent to share contact details with the research team. Clinicians will also send recruitment packs to eligible individuals identified from their patient database. Clinicians can also signpost individuals to the trial web page. Posters advertising the trial will be placed in the patient waiting areas of these clinics.

An online social media recruitment strategy will be used including adverts targeting charities and patient groups.

- Blesma, the limbless veterans

- LimbPower

- Finding Your Feet

- Limbless Association

- Burning Nights - CRPS support

- Defence Medical Welfare Service (DMWS)

- North Cumbria Amputee Society (NCAS)

- Help for Heroes

- Steel Bones

- Clinical Research Network

- NHS regional trusts

Charities will also be asked to include this information in their newsletter where applicable. A trial research page will be launched where people can obtain information about the trial and how to contact the researchers. The information will be that contained within the participant information sheet. Teesside University will advertise the trial on its social media platforms.

At 2PD Ltd., there is a list of people who have PLP who have previously given their consent to have their contact details stored and to be contacted about research they may be eligible for. A reminder letter will be sent two weeks later.

Finally, at Teesside University there is a list of people who have PLP who have previously given their consent to have their contact details stored and to be contacted about research they may be eligible for. A reminder letter will be sent two weeks later.
