# Supplementary material for: Efficacy of a novel sensory discrimination training device for the management of phantom limb pain: protocol for a randomised placebo-controlled trial
Source: BMJ Open. 2025 Nov 9;15(11):e101657. doi: 10.1136/bmjopen-2025-101657 (PMC12598989; doi:10.1136/bmjopen-2025-101657)
Supplement: online supplemental file 3 [file bmjopen-15-11-s003.docx]

**APPENDIX 3**

**Data Monitoring (Ethics) Committee (DMEC) and Trial Steering Committees Responsibilities**

Full charters available by request.

Specific Responsibilities of the DMEC

The DMEC’s responsibilities include, but are not restricted to:

• review and approve the Protocol prior to Governance and Ethics applications

• review and approve any Amendments that may be sought, prior to Governance and Ethics applications being submitted for those

• review and approve the Publication Policy

• review and monitor safety reports and unblinded data

o monitoring for any evidence of intervention harm/issues and futility

• monitor data quality, including completeness

• monitor recruitment, retention, and follow-up rates

o with particular respect to a-priori sample size estimates

• monitor evidence for indications of need for any modifications to the Analysis Plan

• monitor continuing appropriateness of patient information

• assess the impact, importance and relevance of any external evidence that may arise during the Trial

• monitor compliance with DMEC recommendations

• make recommendations to the TSG and PMG regarding continuation (or otherwise) of the Trial

Specific Responsibilities of the TSC

The TSCs responsibilities include, but are not restricted to

• providing expert oversight of the trial and advice to the PMG, the Sponsor, the funder, and relevant Regulatory bodies (as appropriate), on all aspects of the trial.

• review and monitor

o adherence to the protocol and the terms of the Ethical and Governance Approvals/Permissions obtained

o confidentiality of all trial information that is not already in the public domain

o progress of the trial

o reports submitted by the DMEC and PMG prior to each meeting

• consideration of any proposed protocol amendments and providing advice to the CI, sponsor and funder regarding such amendments
