# Supplementary material for: Efficacy of a novel sensory discrimination training device for the management of phantom limb pain: protocol for a randomised placebo-controlled trial
Source: BMJ Open. 2025 Nov 9;15(11):e101657. doi: 10.1136/bmjopen-2025-101657 (PMC12598989; doi:10.1136/bmjopen-2025-101657)
Supplement: online supplemental file 4 [file bmjopen-15-11-s004.docx]

**APPENDIX 4**

**Informed Consent Documents**

**Informed Consent Form**

**Study Title:** Sensory retraining for phantom limb pain

**Researchers:** Professor Cormac Ryan, Ms Sarah Oatway, Dr Andrew Graham, Mrs Sally Smith, Dr Deepak Ravindran, Professor Alan Batterham, Dr Alasdair Macsween, Professor Denis Martin

**HRA reference number:** 23/NS/0085

| 1. | I confirm that I have read and understood the Participant Information Sheet (version 5.5  dated 07.05.24) for this trial. I have had the opportunity to consider the information, ask questions and have had my questions answered satisfactorily. |
| --- | --- |
| 2. | I understand that taking part is voluntary and that I can change my mind at any time before the last data point for the last person is collected, without giving any reason. |
| 3 | I give permission that my GP is told that I took part in this study. |
| 4. | I agree that I may be contacted by email, SMS text message, phone or video call on the email address and telephone number I will supply, and that the personal information needed for that to happen may be shared with SMART-TRIAL and accessed and processed as detailed in the Participant Information Sheet (version 5.5 dated 07.05.24) for this trial. |
| 5. | I agree that my individual results in the trial may be published as an individual case-study where I will not be identified. |
| 6. | I understand that data collected during this trial may be looked at by individuals from the research team, from regulatory bodies or from the NHS Trust, where it is relevant to my taking part in this research. I give permission for these individuals to have access to that data. |
| 7. | I confirm that it is impossible that I could be, or could ever get, pregnant. Or if this is not the case, I agree to undergo a pregnancy test and/or use contraception as described in the PIS. |
| 8. | I agree to take part in this trial. |

Patient Information Sheet and Key Fact Summary Sheet available by request.
