# Supplementary material for: Efficacy of a novel sensory discrimination training device for the management of phantom limb pain: protocol for a randomised placebo-controlled trial
Source: BMJ Open. 2025 Nov 9;15(11):e101657. doi: 10.1136/bmjopen-2025-101657 (PMC12598989; doi:10.1136/bmjopen-2025-101657)
Supplement: online supplemental file 5 [file bmjopen-15-11-s005.docx]

**APPENDIX 5**

**Secondary Outcome Measures**

The following secondary outcomes are specified at the 3-week timepoint (immediately post intervention):

- Overall Pain Intensity Score: Visual Analogue Scale (100mm)^48^
- Frequency adjusted pain score: (0-100)
- General Subjective Outcome Score (GSOS)^24^

*Overall Pain Intensity Score: Visual Analogue Scale (100mm)*

Participants will be asked to rate their PLP intensity over the last week using a pain visual analogue scale consisting of a 100-mm line with anchor statements of 0 representing no pain and 100 the worst pain imaginable. The participant will be asked to place a mark through the line to represent their pain.^48^

*Frequency adjusted pain score: (0-100)*

Participants will be asked to record the frequency of PLP episodes they have experienced over the past week from 1 = all the time to 5 = Once a day or less per week. The overall pain score will be divided by the frequency value to create a composite frequency adjusted pain score ranging from 0-100.

*General Subjective Outcome Score (GSOS)*

Overall perception of outcome will be measured using a Global Assessment Scale. Participants will be asked to rate their level of improvement choosing one of six options, ranging from "worse" to "completely better".^24^

*Exploratory Outcomes*

No inferences will be drawn from these outcomes.

- SF-MPQ-2 total score at the 3-month time point
- TAPES (modified)^16 17^  at 3-weeks and 3-months
- Quality of life [EQ-5D-5L]^9 26^ at 3-weeks and 3-months
- Sleep Disturbance [the PROMIS Short Form v.1.0 – Sleep Disturbance 4a questionnaire]^18^ at 3-weeks and 3-months
- Participant satisfaction ^38^ at 3-weeks and 3-months
- Device Usability [an adapted version of the 10 question System Usability Scale (SUS)].^5^ at 3-weeks
- Study Diary of device and medication usage - Daily
- Concordance with Protocol
- Success of blinding at 3-months

SF-MPQ-2

The McGill Pain Questionnaire revised (SF-MPQ-2) (Dworkin et al., 2009b). 22 items/ pain descriptors across 4 pain sub-scales/ domains: continuous, intermittent, neuropathic, and affective. Participants rate each item on an 11-point (0-10) scale, where 0 = none and 10 = worst possible pain. The mean of the 22 items provides the SF-MPQ-2 total score. Two or more missing responses on any sub-scale results in an invalid outcome.

*TAPES (modified)*

Participants will be asked to complete a modified version of the Trinity Amputation and prosthetics evaluation scale (TAPES scale).^16 17^ This questionnaire records information about the participant’s amputation history, their levels of pain and their level of functioning. The TAPES has been modified in that it only asks about issues specific to PLP, residual limb (or stump) pain, and phantom sensations. Additional questions will be asked upon completion of the TAPES questionnaire. These yes/ no questions will identify if the patient experiences telescoping, which is a phenomenon where the phantom limb is perceived as shrunken so that the foot/hand feels like it is attached directly to the end of the residual limb, and further questions to explore residual limb sensitivity and neuroma history and establish if the participant feels that they have active control of the phantom limb or if it is frozen. There are 22 questions in total. Participants’ age in years, sex, employment status, ethnicity, socioeconomic status (Index of multiple deprivation score calculated from post code) will be recorded within this questionnaire.

*Quality of life*

The EQ-5D-5L is a brief easy to use validated measure of quality of life.^9 26^ The EQ-5D-5L contains five items related to mobility, self-care, usual activities, pain/ discomfort, and anxiety/ depression. Each dimension has five levels: no problems, slight problems, moderate problems, severe problems, and extreme problems. Participants indicate their health state by selecting the most appropriate statement in each of the five dimensions, resulting in a 1-digit number. The digits for the 5 dimensions can be combined into a five-digit number describing the participant’s health state. The EQ-5D-5L value set for England published by Devlin *et al*^9^ will be used to calculate the index score. There is also a 100-mm visual analogue scale for rating general health, labelled from ‘the worst health you can imagine’ to ‘the best health you can imagine’.

*Sleep Disturbance*

Participants will be asked to complete the PROMIS Short Form v.1.0 – Sleep Disturbance 4a questionnaire. This is a self-reported questionnaire reporting perceptions of sleep quality, sleep depth, and restoration associated with sleep. It assesses sleep disturbance over the past seven days. There are four questions, each containing a 5-point Likert Scale, ranging from Very Poor to Very Good. The questions are then combined to create a score out of 100.^18^

*General Subjective Outcome Score (GSOS)*

Overall perception of outcome will be measured using a Global Assessment Scale. Participants will be asked to rate their level of improvement choosing one of six options, ranging from "worse" to "completely better".^24^

*Participant satisfaction*

*P*articipants satisfaction with their device will be investigated using a six-item rating scale based upon the NHS Friends and Family Test ^38^ Participants will be asked to answer the question, *"How likely are you to recommend the SP1X to friends and family if they had phantom limb pain?"* by choosing one of six options, ranging from "extremely likely" to "extremely unlikely".

*Device Usability*

To investigate device usability, at the 3-week point, all participants will be asked to complete an adapted version of the 10 question System Usability Scale (SUS).^5^ This is a commonly used tool to assess the useability of medical devices. It is a 10 item questionnaire with five responses for each item ranging from Strongly agree to Strongly disagree

*Study Diary*

In addition to the data collection time points of 3-weeks and 3-months, participants will be asked to report on their daily medication usage over the duration of the trial in a study diary. The participant will be sent a daily notification by the SMART-TRIAL platform to remind them to complete the study diary for the three-week treatment period. The diary will ask for the frequency, dosage, and class of medication taken each day. In addition, patients will be asked to rate their PLP on a scale from 0-10 each day, the frequency of those pain episodes, and to record how many minutes they used the device each day.

Process Measures

*Concordance with Protocol*

The frequency and duration of use of the devices will be automatically captured by the device and supplemented by patient use documentation in the daily study diary. This will be used to assess compliance with the protocol.

*Success of blinding*

To investigate the success of the participant blinding procedures the participants will be asked to make a judgement, after completing the second set of outcome measures and before the blinding is broken. Participants will be asked *Which of the following devices do they believe the received?* And then *How confident they are of this on a 0-100 scale with 0 being not at all confident and 100 being completely confident?*

Regarding the primary outcome measure of pain - the SF-MPQ-2, which uses a 0-10 scale, a minimally clinically important difference between groups of 1 point will be used.
