# Supplementary material for: Efficacy of a novel sensory discrimination training device for the management of phantom limb pain: protocol for a randomised placebo-controlled trial
Source: BMJ Open. 2025 Nov 9;15(11):e101657. doi: 10.1136/bmjopen-2025-101657 (PMC12598989; doi:10.1136/bmjopen-2025-101657)
Supplement: online supplemental file 6 [file bmjopen-15-11-s006.docx]

**APPENDIX 6**

**Reporting of Adverse Events and Adverse Device Events**

All discovered/ disclosed Adverse Events (AEs) and Adverse Device Events (ADEs) will be recorded by the RA on the EDC platform, which follows ISO14155:2020 Adverse Event and MEDDEV 2.7/3 reporting processes. AEs and ADEs will be reported to the CI and the Sponsor’s representative by automatic notification from the EDC. The CI will send the Chair of the DM(E)C all AE and ADEs not later than within two working days of discovery/ disclosure. The CI will report non-person identifiable summary information on all ADEs to the Manufacturers’ Representative on the TSG immediately but not later than within two calendar days of becoming aware of them. AEs and ADEs will be processed and evaluated by the CI, the Sponsors representative, and the Chair of the DM(E)C in accordance with ISO:14155.2020 sections 7.4.2/3, 9.2.5 and 10.8. The CI will submit a non-person identifiable summary of all AEs and ADEs to the Chair of the Trial Steering Group (TSG) for inclusion as a Standing Item on the TSG Agenda. Non-person identifiable summary statistics for AEs and ADEs may be included in Disseminations and/or further Regulatory/Licensing Applications.

Reporting of SAE, SADEs, USADEs

All SAEs, SADEs, and USADEs will be recorded immediately but preferably not later than within two calendar days of discovery/ disclosure on the EDC platform. The system will automatically notify the CI and the Sponsor’s Representative of all SAEs, SADEs, and USADEs, prompting actions to be carried out on the system. These actions accord with MEDDEV 2.3/7. The CI will report all SAEs, SADEs, and USADEs to the Chair of DM(E)C, the main HRA RES REC, and the MHRA (National Competent Authority) immediately but not later than within two calendar days of becoming aware of them, using the reporting template in the Appendix to MEDDEV 2.7/3.The CI will report non-person identifiable summary information on all SADEs, and USADEs to the Manufacturers’ Representative on the TSG immediately but not later than within two calendar days of becoming aware of them. The CI will submit a non-person identifiable summary of all SAEs, SADEs, and USADEs to the Chair of the Trial Steering Group (TSG) for inclusion as a Standing Item on the TSG Agenda. Non-person identifiable summary statistics for SAEs, SADEs, and USADEs may be included in disseminations and/or further Regulatory/Licensing Applications.

Reporting of Device Malfunctions or Failures

Any device malfunction or failure, including use errors, will be recorded, and reported by the RA using the EDC platform, which follows ISO14155:2020 Adverse Event and MEDDEV 2.7/3 reporting processes (Appendix 60). Where an event is not associated with an Adverse Event, for example the device will not turn on at first use, this will be clearly categorised as ‘Device Malfunction’ (Non AE). The CI will report these to the Sponsor's Representative and Manufacturer for evaluation and investigation, regardless of whether or not an adverse event was associated with the device malfunction or failure. Non-person identifiable summary statistics for device malfunction or failures may be included in disseminations and/or further Regulatory/Licensing Applications
