# Supplementary material for: Efficacy of a novel sensory discrimination training device for the management of phantom limb pain: protocol for a randomised placebo-controlled trial
Source: BMJ Open. 2025 Nov 9;15(11):e101657. doi: 10.1136/bmjopen-2025-101657 (PMC12598989; doi:10.1136/bmjopen-2025-101657)
Supplement: online supplemental file 7 [file bmjopen-15-11-s007.docx]

**APPENDIX 7**

There will be two parallel groups, an intervention group, and a placebo group

|  | Item |  |  |
| --- | --- | --- | --- |
| 1 | Brief Name | Intervention Group | Placebo Group |
| 2 | Why | After amputation many amputees experience Phantom Limb Pain (PLP). Sensory Discrimination Training (SDT) is a nonpharmacological intervention which aims to alter cortical representation so that it is more aligned to the actual limb. SDT involves stimulating a number of spatially close, but distinct, areas on the residual limb, the patient judging which area was stimulated, and then receiving feedback on that judgement. To date, SDT requires a clinician, or carer, to administer the stimulation. To overcome this practical challenge the *SP1X* has been developed. It is a fully automated, interactive SDT device which is self-administered by the patient. To date there have been no studies investigating the efficacy of this device. The primary aim of this trial will be to investigate the efficacy of a the *SP1X* for the management of PLP | |
| 3 | What  (Materials) | SP1X  The device is a sensory discrimination training device which can be used by a participant independently following brief training. A patch holding twelve equally spaced electrodes is placed over the residual limb adjacent to, but not over, the healed wound. This pad is connected, via Bluetooth, to a separate tablet device running an Android 10+ app designed to deliver the sensory discrimination training programme. The programme sends a signal to the pad to deliver an electrical current to the electrodes | SP1XP  Those allocated to the placebo group will be provided with the placebo, non-active *Sham* device. This will look identical to the intervention device, but it will not deliver stimuli to the participant |
| 4 | What  (Procedures) | The *SP1X* stimulates the skin via one of the electrodes using either a single pulse, or a rapid burst of pulses. A single pulse is like a long single tap on the skin, while a burst of pulses is like a group of short rapid taps, in sequence. The participant interacts with the device via a touchscreen. Immediately upon delivery of a stimulation by the device, the touch screen display presents the following questions. Q1. *Which electrode* (location) *was stimulated? Q2. Was a ‘continuous’ pulse or a ‘burst’ of pulses given* (stimulation type)*?* The participant answers using a touch screen with a range of answer options dependent on the level of challenge the device is set. The display then tells the participant whether or not their response was correct. If correct, a new stimulus location/ type is delivered, and the same questions presented. If incorrect, the user is informed what the correct response was and the previous stimulation is repeated, to give immediate feedback, before changing stimulus location/ type.  The *SP1X* has six different levels of difficulty. The easiest levels use fewer electrodes (e.g., 2 electrodes), which means that the stimuli are delivered further apart from one another, and the location of the stimulus is thus easier to identify. The moderate levels use between 4, 6, 8 and 10 electrodes, and the hardest 12. With each level increment, the number of electrodes increases; thus, the distance between stimuli gets smaller and the location becomes more difficult to identify. As the participant improves to a point where they are consistently judging the stimuli correctly (90% of the time), the device will automatically increase the difficulty level so that the participant progresses | There is no interaction with the placebo device; that is, there is no Q&A element, feedback, nor response-dependent progression. Participants in this group will be informed that the device works to facilitate cortical reorganisation, which occurs at a wholly unconscious level. As a result, they need not engage with the device after it has been turned on and are free to do *other things* while the stimulation is underway, for example, read a book or watch TV. In order to avoid confounding variables, *other things* will be restricted to seated activities, as the participants in the intervention group will need to be seated whilst using the *SP1X* device |
| 5 | Who | The intervention is self-administered by the patient themselves. However, the research assistant (RA) who is a state registered physiotherapist, will provide instruction on how to use the device. | |
| 6 | How  (Mode) | The Trial is fully decentralised with participants taking part from their own homes, using an Electronic Data Capture tool to complete all trial documentation. All communications between trial participants and the research team will be via email, SMS messages, telephone, or video conferencing platforms.  During an online video call the participant will be instructed how to set up and use the device by the research assistant (RA). The RA will observe the first full treatment session (60 minutes) and (up to 30 minutes of) the second full treatment session and provide any additional advice needed. Participants in both groups will be asked to use the device for 60 minutes each day, as one block, or as multiple shorter sessions of ≥20 minutes duration on at least 15 days of the 21-day treatment period. They will be asked to spread out the use of the device, over the treatment period and to record their device use in a study diary | |
| 7 | Where | Participants use in their own homes | |
| 8 | When & How Much | During an online video call the participant will be instructed how to set up and use the device by the research assistant (RA) who is a state registered physiotherapist. The RA will observe the first full treatment session (60 minutes) and (up to 30 minutes of) the second full treatment session and provide any additional advice needed. Participants in both groups will be asked to use the device for 60 minutes each day, as one block, or as multiple shorter sessions of ≥20 minutes duration on at least 15 days of the 21-day treatment period. They will be asked to spread out the use of the device, over the treatment period and to record their device use in a study diary.  During the three-week intervention period all participants will receive daily email and SMS reminders from the EDC platform, reminding them to use the device and complete the device usage and pain medication diaries. The participants will also receive video calls at day 4, 7 and 14 of the trial to check if there are any issues (e.g., a device malfunction) and answer any questions that the participant might have regarding the device. Video calls will also take place after 3 week and 3-month outcome data has been collected using the EDC platform.  All video call appointment times will be subject to minor deviations to facilitate data collection and minimise patient burden due to unforeseen circumstances. For example, if a participant is unwell or unable to meet with the RA precisely at 3 months, for whatever reason, the meeting can be arranged for the next available day, to mirror clinical practice. Any deviations in outcome measure timing will be recorded on the EDC platform | |
| 9 | Tailoring | N/A | |
| 10 | Modifications | N/A  Any amendments to the approved protocol will be recorded in the Trial Master File | |
| 11 | How Well  (Fidelity) | The CI will undertake a checklist fidelity check for all participants during the treatment period of participation. | |
| 12 | Actual  (Adherence) | N/A recruitment ongoing.  Adherence will be reported as a secondary outcome measure. | |

TIDieR Table for the Intervention and Placebo Groups
