# Supplementary material for: Efficacy of a novel sensory discrimination training device for the management of phantom limb pain: protocol for a randomised placebo-controlled trial
Source: BMJ Open. 2025 Nov 9;15(11):e101657. doi: 10.1136/bmjopen-2025-101657 (PMC12598989; doi:10.1136/bmjopen-2025-101657)
Supplement: online supplemental file 8 [file bmjopen-15-11-s008.docx]

**APPENDIX 8**

**Topic prompts for Semi Structured Interviews**

To be conducted on video call and transcribed using TEAMs software.

Questions for Semi Structured Interviews.

1. Please tell me how your amputation came about, and your subsequent experience of phantom limb pain. (icebreaker question)

Prompt – example - Pain medication/healthcare usage – who dealt with your pain management (GP/Consultant/Physio etc)

1. Overall, what did you think about the device? (usability)
2. Are the instructions for use clear?
3. Did you find it comfortable to wear?
4. How do you feel about the time commitment for treatment?
5. Was the hand-held device easy to use?
6. Any barriers to you using the device?
7. How did you use the device, and why did you use it in this way?

Prompt – example – why chose to use in one 90 minute session rather than multiple 10 minute sessions.

1. Are there any improvements you would make to the device?
2. Overall Design
3. ‘Wearability’ (device comfort)
4. Software – Interaction with device – anything missing i.e. recognition for level progression
5. Were the text messages we sent to you, to remind you to use the device and to complete your study diaries useful, or not? And why? (Prompt – did these messages influence how you used the device?)
6. Do you have any other comments you would like to make about the device, or taking part in in the trial in general?
