# Supplementary material for: Efficacy of a novel sensory discrimination training device for the management of phantom limb pain: protocol for a randomised placebo-controlled trial
Source: BMJ Open. 2025 Nov 9;15(11):e101657. doi: 10.1136/bmjopen-2025-101657 (PMC12598989; doi:10.1136/bmjopen-2025-101657)
Supplement: Supplementary file 9 [file bmjopen-2025-101657.reviewer_comments.pdf]

PEER REVIEW HISTORY

BMJ Open publishes all reviews undertaken for accepted manuscripts. Reviewers are asked to complete a checklist review form and are provided with free text boxes to elaborate on their assessment. These free text comments are reproduced below.

ARTICLE DETAILS

Title (Provisional)

Efficacy of a novel sensory discrimination training device for the management of phantom limb pain: protocol for a randomised placebo-controlled trial

Authors

Oatway, Sarah; Martin, Denis; Graham , Andrew; Batterham, Alan; MacSween , Alisdair; Smith , Sally; Ravindran, Deepak; Ryan, Cormac

---

VERSION 1 - REVIEW

**Reviewer**	**1**
**Name**	**Guémann, Matthieu**
**Affiliation**	**Université d'Orléans**
**Date**	**20-Jul-2025**
**COI**	

Dear Authors,

Thank you for the opportunity to read and evaluate your manuscript.

Overall, this is a well-written protocol. I appreciate the clarity of the structure, and I recognise the importance placed on reproducibility and transparency in data management. That said, I have a number of suggestions which may help to improve both the clarity and completeness of the manuscript.

Major Comments:

Intervention procedure and device

My main concern relates to the description of the intervention and the device used. Key information regarding the device—such as the type of device, its functioning, and the rationale for its use—is absent from the main text. At present, only the training procedure is briefly described in Appendix 7, which is insufficient given the central role of the intervention and its underlying technology.

I understand that detailed information about the device may be restricted due to industrial or proprietary concerns. However, it would be important to at least introduce the electro-tactile technology in the Introduction, with a short paragraph discussing its relevance for discrimination training, including comparisons with other techniques such as vibrotactile stimulation (please refer to my comments in the annotated Word document).

Furthermore, the Intervention section should be expanded, integrating some of the content currently found in Appendix 7. This includes what participants will be asked to do, how they will interact with the device, and how the intervention might evolve for each group.

Minor Comments

Abstract:

• The required PLP level for inclusion should be stated.

• There is a repetition between the "Methods and Analysis" and "Intervention" sections:

“The first and second treatment sessions will be observed via video call to provide set-up guidance and any additional advice needed”

“The RA will observe the first and second treatment sessions and provide any additional advice needed”

Please revise this to avoid redundancy and use the space to elaborate on other aspects of the methodology.

• The main statistical analysis should be described more precisely.

Introduction:

• The introduction is generally well-structured. However, some contextual elements are missing.

• The limitations of non-pharmacological interventions are briefly mentioned, but this section could be strengthened to better justify the study. For example, consider discussing the limitations of established approaches such as mirror therapy or graded motor imagery, and the heterogeneity in clinical practice (see comments in the document).

• A paragraph discussing the value of electro-tactile devices is also needed, to clarify the rationale for choosing this technology.

Methods

• The manuscript refers to upper-limb amputees "above the elbow". I'd like to be sure that this is not “above the wrist” for the upper limb as major amputation starts from here.

• The inclusion and exclusion criteria could be better structured—consider presenting them in a table for clarity.

• The recruitment strategy deserves a dedicated paragraph and could benefit from clearer organisation (please refer to my comment in the document).

• Sample size: It would be helpful to refer to a relevant MCID (Minimal Clinically Important Difference), if available.

• Intervention: As noted earlier, this section should include more detail from Appendix 7.

• Including a picture of the device would improve understanding.

• Statistical analysis: In addition to p-values, consider including effect size estimates, which are more informative for interpreting the results.

• The manuscript mentions a per-protocol analysis. Please provide justification for this choice and explain why an intention-to-treat (ITT) approach was not adopted.

• It would be helpful to include or describe the template for the semi-structured interviews.

Other points

• The checklist refers to strategies to improve adherence being reported on page 3. Could the authors elaborate on what is done specifically during the 3-week intervention to support adherence?

• The follow-up at 3 months is relatively short. Although I understand that this may be due to the constraints of the thesis timeline, a longer-term follow-up (e.g., 6 months or 1 year) would have added valuable insight.

Thank you again for the opportunity to review your work. I hope these comments are helpful as you continue developing this promising protocol.

Best regards,

****** The reviewer provided a marked copy with additional comments. Please contact the publisher for full details. ******

Reviewer 2

Name McGuire, Brian

Affiliation National University of Ireland, Galway, Ireland, School of Psychology & Centre for Pain Research

Date 30-Jul-2025

COI

This protocol paper describes the intended evaluation of a sensory discrimination device for the treatment of phantom limb pain (PLP). PLP is common and often intractable so novel approaches to treatment are important. The proposed design is an RCT with 50 per group. The treatment will be self-administered at home. The device itself has not been tested in a clinical trial but there is minimal likelihood of harm as the device merely applies a light electrical current to the residual limb. The background and theory of mechanism of action (cortical reorganisation) have been described clearly and with sufficient detail. Overall, the protocol has a high level of detail and a high degree of rigour. I had some minor comments and queries as follows:

1. The duration of pain is not specified - usually trials target chronic pain, that is, pain that has been present for at least 3 months.

2. In the inclusion criteria, a minimum level of pain of at least 4/10 for at least two days per week is specified - how was this chosen? (4 is an arbitrary number and would typically represent medium-low pain intensity).

3. The inclusion criteria are above ankle or above elbow amputation - I wondered why not above wrist rather than elbow?

4. Pregnant or possibly/intending tp be pregnant women are excluded - what is the rationale for that?

5. The effect size section on page 4 line 5 refers to a change at "follow-up" at 3 weeks. Do you mean post-intervention (3 weeks) or follow-up (3 months)?

6. The sample size estimate is based on an effect size of 1 point change between groups on McGill short-form. Please justify this as a clinically meaningful change (at face value, one point change means approximately 10% improvement).

7. Provide some text to briefly explain outcome measures/domains e.g. TAPES

VERSION 1 - AUTHOR RESPONSE

Reviewer: 1
Dr. Matthieu Guémann, Université d'Orléans

****** The author provided a marked copy with additional comments. Please contact the publisher for full details. ******

N.B., *We have added these comments and our responses to this document, using comment ref number (i.e. [R11]) and page number from the attached file, for ease of reading.*

Dear Authors,
Thank you for the opportunity to read and evaluate your manuscript.
Overall, this is a well-written protocol. I appreciate the clarity of the structure, and I recognise the importance placed on reproducibility and transparency in data management. That said, I have a number of suggestions which may help to improve both the clarity and completeness of the manuscript.

Major Comments:
Intervention procedure and device

My main concern relates to the description of the intervention and the device used. Key information regarding the device—such as the type of device, its functioning, and the rationale for its use—is absent from the main text. At present, only the training procedure is briefly described in Appendix 7, which is insufficient given the central role of the intervention and its underlying technology.
I understand that detailed information about the device may be restricted due to industrial or proprietary concerns. However, it would be important to at least introduce the electro-tactile technology in the Introduction, with a short paragraph discussing its relevance for discrimination training, including comparisons with other techniques such as vibrotactile stimulation (please refer to my comments in the annotated Word document). **([R18] "references should be add to this affirmation. Some concern about the material can be introduce and different option are explored (electric, or virbotactile)." pg.3)**
Response: Thank you, we have added acknowledgement of vibrotactile stimulation as an alternative to electrical stimulation to deliver SDT with appropriate citations (pg. 2)

Furthermore, the Intervention section should be expanded, integrating some of the content currently found in Appendix 7. This includes what participants will be asked to do, how they will interact with the device, and how the intervention might evolve for each group. (**[R120] "some part of it should be include in the main text especially about what material and how it works" pg. 22)**
Response: Thank you for this comment. We have added the following brief explanation of the device for clarity, with the inclusion of a picture (pg.4)

Intervention
There will be two parallel groups, an intervention group (SP1X) and a placebo group (SP1X7). Both the intervention and placebo device**, which are identical in appearance,** will be provided to participants for use in their own homes. **A picture of the device is provided in fig.1.**
Fig.1

During an online video call the participant will be instructed how to set up and use the device by the RA (an HCPC registered Physiotherapist). **Both devices have an electrode patch which is placed on to the residual limb. This pad is connected, via Bluetooth, to a separate tablet device running an Android 10+ app designed to deliver either an interactive SDT programme or a non-interactive programme**.

Minor Comments
Abstract:
1) The required PLP level for inclusion should be stated**. ([R11]"level of PLP should be precise. Number of participants have to be develop in the method section"'pg. 1)**
Response: We have added to the abstract section as follows:
*Eligible, consenting individuals with PLP **(intensity rated as ≥4 on a 0-10 scale)** (n=100) will be randomised to receive the SP1X device (Intervention group) or a placebo device SP1X7 (placebo group).{pg.1)*
2) There is a repetition between the "Methods and Analysis" and "Intervention" sections:
"The first and second treatment sessions will be observed via video call to provide set-up guidance and any additional advice needed"
"The RA will observe the first and second treatment sessions and provide any additional advice needed"

Please revise this to avoid redundancy and use the space to elaborate on other aspects of the methodology.
([R12] "their is a repetition with the paragraph "intervention" this should be remove to better clarity" pg. 1)
Response: On checking the manuscript, we are confident that the repetition occurs between the methods and analysis section of the abstract section, rather than main Methods and Analysis section of the article, and the intervention sections of the article. We have not removed this repetition as we believe it is necessary for understanding this protocol paper if the reader were to only read the abstract.

3) The main statistical analysis should be described more precisely.**([R13] "what will be the main test" pg.1)**
Response: We have added the following for further clarity in this section.
Statistical analysis will be carried out by a blinded statistician ***(ANCOVA model conditioning on the baseline and stratification factors).*** *Semi-structured interviews will be carried out with a sub-sample (n=10-15) of intervention group participants. (pg.1)*

[R14]' eligible criteria should appear in the method section'pg.2 – *Response – we have reviewed this and can confirm that that description is of a strength of the study rather than an eligibility criteria, which are detailed in the methods section.*

Introduction:
• The introduction is generally well-structured. However, some contextual elements are missing.

4) The limitations of non-pharmacological interventions are briefly mentioned, but this section could be strengthened to better justify the study. For example, consider discussing the limitations of established approaches such as mirror therapy or graded motor imagery, and the heterogeneity in clinical practice (see comments in the document). **([R15] "Some developpement can be done about this limitation and current technics to alievate PL, espeUliaBy MT ou GMI" pg.2)**
Response: Due to the word count and therefore limited space to include a discussion we have supported our statement with citation of a peer reviewed systematic review of non-pharmacological treatments. We thank you for the papers you have suggested here, which we have also added as citations. (pg. 2)

[R16] "physiopathology"pg.2 - *Response: We have changed the word pathology to* ***pathophysiology*** *(pg. 2)*

[R17] "I would suggest to first present the results of the paper and then the seminal paper of H.Flor that confirm and reinforce the findings." – *Response: We thank you for this suggestion. We have reflected on how we have ordered this paragraph and have respectfully decided to leave the order unchanged.*

5) A paragraph discussing the value of electro-tactile devices is also needed, to clarify the rationale for choosing this technology.
Response: We have addressed this in our previous response to this comment above.

Methods
6) The manuscript refers to upper-limb amputees "above the elbow". I'd like to be sure that this is not "above the wrist" for the upper limb as major amputation starts from here.**[R19] "above elbow and not wrist ?"**
Response: Thank you for noting this, it should read 'above wrist' not above elbow. We have corrected this.

7) The inclusion and exclusion criteria could be better structured—consider presenting them in a table for clarity.
Response: We thank you for your comment regarding the summary of eligibility criteria included here. We have provided the full criteria as an appendix for readers to reference should they wish. We are confident that this provides sufficient information should it be of interest, without distracting from the more salient information within the main body of the article.

[R110] "the sentence can be confusing using at the same time exclusion and include" *– We have removed 'these include' and replaced with 'for example' for clarity.*

[R111]* "*just mention where it is (on th stump)?" *– We have not made a change here, as on reflection, we respectfully believe it is best way to describe the guidance of electrical stimulation for those with metal implants.*

***[R112]* "*including inclusion and exclusion? this can be precise"* -** *We have changed this to inclusion and exclusion on your recommendation.*

8) The recruitment strategy deserves a dedicated paragraph and could benefit from clearer organisation (please refer to my comment in the document).**[R113] "a specific paragraph about the recruitment procedure can be add. And the method should be clarified. Here it seems that you put everything but without any order. Classification can be main between direct and indirect contact"**
Response: Thank you for your suggestion. We have reviewed this and respectfully believe a concise overview is given, with the further inclusion of the full strategy in an appendix. Thus, we have decided to make no change here.

9) Sample size: It would be helpful to refer to a relevant MCID (Minimal Clinically Important Difference), if available. **([R114] "ok but for this data, you should rely to the MCID: (I assume you know the reference)"pg.5)**
Response: We thank you for this comment. We have added the following statement to the text: :
An MCID for pain for people with PLP has not been definitively established. However, for chronic pain in general, according to the IMMPACT recommendations, a reduction in pain of 10-20% can be considered to represent a "minimally important" clinical change (Smith et al. 2020). This is in concordance with recent NICE guidelines for chronic pain which have identified "the use of or 10% of a scale" as one appropriate option for between group differences in pain (NICE, 2021).

Additionally, thank you also for raising the article by Ryan et al. 2024. This paper was published by individuals from the authorship of the submitted paper and the data from it was used to estimate the required sample size for this study. We have now referenced this paper to acknowledge this.

[R115] "the stratification is very important to have the possibility to explore the data in details."
Response: We agree with your comment

10) Intervention: As noted earlier, this section should include more detail from Appendix 7.
Response: Please refer to our previous response to this point made earlier.

11) Including a picture of the device would improve understanding
Response: We agree and have added a picture to improve understanding in the intervention section (pg.4)

12) Statistical analysis: In addition to p-values, consider including effect size estimates, which are more informative for interpreting the results. **[R116] "the p-value is not the best indicator of the strenght of the result. I would advice to use an estimation approach: https://doi.org/10.1016/j.jphys.2021.12.001"**
Response: Please note that we have stated that 95% confidence interval is stated alongside the p-value (pg.6) as follows:
The primary endpoint will be the estimate of the effect of the intervention at 3 weeks. We shall present the point estimate together with 95% confidence interval and P-value, via t-based inferences using Satterthwaite degrees of freedom.

We have added the following sentence (pg.6): The confidence interval provides a plausible range of effect sizes compatible with the data, model, and underlying statistical assumptions, and represents an estimation approach.

We wish to retain the presentation of the P-value for completeness, consistent with a frequentist statistical analysis.

13) The manuscript mentions a per-protocol analysis. Please provide justification for this choice and explain why an intention-to-treat (ITT) approach was not adopted.**[R117] "why don't you use an ITT analysis"**

Response: Please note that we originally described an intention to treat analysis for the primary outcome measure (pg.6) as follow:
An intention-to-treat analysis (primary) and a per-protocol analysis (secondary) will be undertaken. In the per-protocol analysis the participant will be required to have undertaken at least 10 sessions of sensory discrimination training totalling (600 minutes) to be considered suitable for inclusion in the analysis. The primary analysis will be carried out by a statistician blinded to group assignment.

[R118] "is the sentence finished?"
Response: Yes, the sentence is finished

14) It would be helpful to include or describe the template for the semi-structured interviews. **[R119]** "**Could the semi- strcutured interview be presented in an appendix file for transparency?"**
Response: Thank you for your comment, as suggested, we have added the template for the semi-structured interviews as an appendix (8) for reference.

Other points
15) The checklist refers to strategies to improve adherence being reported on page 3. Could the authors elaborate on what is done specifically during the 3-week intervention to support adherence?
Response: Thank you for your comment, to which we have added a sentence within the manuscript to concord with the statements made within the appendix (pg.4)
Participants in both groups will be asked to use the device for 60 min/day, as one block, or as multiple shorter sessions of ≥20 minutes duration on at least 15 days of the 21-day treatment period**. Participants will receive support phone calls alongside daily texts to aid adherence to the protocol**. They will be asked to spread out the use of the device, over the treatment period and to record their device use in a study diary.

16) The follow-up at 3 months is relatively short. Although I understand that this may be due to the constraints of the thesis timeline, a longer-term follow-up (e.g., 6 months or 1 year) would have added valuable insight.
Response: Thank you for your comment, we agree with you that a longer follow up period would have been preferable. Multiple factors, which include project funding and logistical considerations, contributed to the decision to follow up after 3 months.

Thank you again for the opportunity to review your work. I hope these comments are helpful as you continue developing this promising protocol.

Best regards,

Reviewer: 2
Dr. Brian McGuire, National University of Ireland, Galway, Ireland
Comments to the Author:
This protocol paper describes the intended evaluation of a sensory discrimination device for the treatment of phantom limb pain (PLP). PLP is common and often intractable so novel approaches to treatment are important. The proposed design is an RCT with 50 per group. The treatment will be self-administered at home. The device itself has not been tested in a clinical trial but there is minimal likelihood of harm as the device merely applies a light electrical current to the residual limb. The background and theory of mechanism of action (cortical reorganisation) have been described clearly and with sufficient detail. Overall, the protocol has a high level of detail and a high degree of rigour. I had some minor comments and queries as follows:

1. The duration of pain is not specified - usually trials target chronic pain, that is, pain that has been present for at least 3 months.
Response: Thank you for you comment regarding chronic pain. In terms of eligibility, we have stated that there be a presence of PLP only, be it acute or chronic. This study focusses on phantom limb pain rather than acute or chronic phantom limb pain. However, we appreciate that chronicity of pain can influence outcomes, thus we are stratifying patients based upon the chronicity of their symptoms.

2. In the inclusion criteria, a minimum level of pain of at least 4/10 for at least two days per week is specified - how was this chosen? (4 is an arbitrary number and would typically represent medium-low pain intensity).
Response: We wish to include people with pain that would be considered moderate to severe rather than mild, so that there would be a clinically relevant need for an intervention. While there is no definitive value that would typically represent the transition from mild to moderate pain previous authors have proposed pain of 1-3 out of 10 as mild and 4 to 6 as moderate and 7+ as severe (Zelman DC, et al. Identification of cut-points for mild, moderate and severe pain due to diabetic peripheral neuropathy. Pain. 2005 May;115(1-2):29-36. doi: 10.1016/j.pain.2005.01.028. PMID: 15836967.) Thus, we used a minimum of 4/10 as our eligibility criteria.

3. The inclusion criteria are above ankle or above elbow amputation - I wondered why not above wrist rather than elbow?
Response: This was an error; it should read as above wrist. This has been corrected in the manuscript on page 3.

4. Pregnant or possibly/intending to be pregnant women are excluded - what is the rationale for that?
Response: Many thanks for this question. We were guided by the following recommendations, for testing in clinical trials and safety of electrical stimulation in pregnancy.
Hma.eu. 2021. Recommendations related to contraception and pregnancy testing in clinical trials. [online] Available at: <https://www.hma.eu/fileadmin/dateien/Human_Medicines/01-About_HMA/Working_Groups/CTFG/2020_09_HMA_CTFG_Contraception_guidance_Version_1.1_updated.pdf> [Accessed 26 April 2021].
Agents, E. (2010) 'Contraindication and Precautions: An Evidence-Based Approach to Clinical Decision Making in Physical Therapy', Physiotherapy Canada, 62(5). The former provides instruction on the inclusion of women who may be pregnant in clinical trials where there is a real or potential risk to the mother/foetus. As this was the first time this specific device was being used with humans the risk was unknown and thus a cautious approach was taken to minimise the risk that pregnant women would take part. The latter provides instruction on contraindications and precautions to use when delivering therapeutic electrical stimulation, as is delivered in this study. We applied the same approach to contraindications and risks to the device in this study as we would to devices such as Transcutaneous Electrical Nerve Stimulation (TENs).

5. The effect size section on page 4 line 5 refers to a change at "follow-up" at 3 weeks. Do you mean post-intervention (3 weeks) or follow-up (3 months)?
Response: Thank you for this, for clarity we have changed this to post intervention as follows:
With an estimated loss to follow-up ***at 3 weeks (post intervention)*** *of 20%, the effective sample size is 80 participants (40 per group in a 1:1 allocation ratio). Our targeted effect size is a difference in* ***post intervention*** *(3-week timepoint) SF-MPQ-2 total score between arms of 1 unit – a moderate clinically relevant effect size.*

6. The sample size estimate is based on an effect size of 1 point change between groups on McGill short-form. Please justify this as a clinically meaningful change (at face value, one point change means approximately 10% improvement).
Response: An MCID for pain for people with PLP has not been definitively established. However, for chronic pain in general, according to the IMMPACT recommendations, a reduction in pain of 10-20% can be considered to represent a "minimally important" clinical change (Smith et al. 2020). This is in concordance with recent NICE guidelines for chronic pain which have identified "the use of or 10% of a scale" as one appropriate option for between group differences in pain (NICE, 2021).

7. Provide some text to briefly explain outcome measures/domains e.g. TAPES
Response: We have provided this supplementary detail in appendix 5 rather than the in the main body of the article due to the limited word count of the article.

VERSION 2 - REVIEW

**Reviewer**	**1**
**Name**	**Guémann, Matthieu**

Affiliation **Université d'Orléans**

Date **10-Oct-2025**

COI

Thanks for letting me assess the revised version of the manuscript. The authors have taken into consideration most of the reviewers' comments, and the overall clarity of the manuscript has improved.

I only have a few minor comments:

Abstract section:

To avoid repetition and a back-and-forth feeling while reading, I suggest that the authors restructure the abstract according to the following sequence: Methods / Intervention / Analysis, instead of Methods and Analysis / Intervention.

I find it easier to describe the intervention before explaining the analysis. In the current abstract, the authors begin to describe the intervention in the first section, then move to the analysis, and return to the intervention later, which makes the reading less fluid.

In the paragraph about the sample size:

The sentence "the use of or 10% of the scale" seems to contain a typo — I assume the word "or" was added by mistake.

There is some repetition between the content of the "Sample size" paragraph and the "Primary and secondary outcome measures" paragraph regarding the MCID. As this concept is already well explained in the "Sample size" paragraph, I suggest that the authors delete the corresponding part in the "Primary and secondary outcome measures" one — specifically, the sentence starting with "A target effect size…".

Acronyms:

Could the authors please reduce the use of acronyms as much as possible? While all abbreviations may be familiar to the authors, they are not necessarily clear to readers, which sometimes makes the reading less comfortable. For example, HCPC is never defined, and PIS, PI, etc., could be clarified. It is often recommended to avoid acronyms in the abstract; they should be used only when absolutely necessary.

Reviewer **2**

Name **McGuire, Brian**

Affiliation **National University of Ireland, Galway, Ireland, School of Psychology & Centre for Pain Research**

Date **10-Oct-2025**

COI

I am of the view that the authors have done an excellent job in addressing my suggestions and requests for clarification.

VERSION 2 - AUTHOR RESPONSE

Reviewer: 1

Dr. Matthieu Guémann, Université d'Orléans
Comments to the Author:
Thanks for letting me assess the revised version of the manuscript. The authors have taken into consideration most of the reviewers' comments, and the overall clarity of the manuscript has improved.
I only have a few minor comments:

Abstract section:
To avoid repetition and a back-and-forth feeling while reading, I suggest that the authors restructure the abstract according to the following sequence: Methods / Intervention / Analysis, instead of Methods and Analysis / Intervention.
I find it easier to describe the intervention before explaining the analysis. In the current abstract, the authors begin to describe the intervention in the first section, then move to the analysis, and return to the intervention later, which makes the reading less fluid.

Response: We thank the reviewer for this comment. On this occasion we have chosen to keep the structure in accordance with the journal guidance as per instructions from the editor. Therefore, no changes have been made.

In the paragraph about the sample size:
The sentence "the use of or 10% of the scale" seems to contain a typo — I assume the word "or" was added by mistake.

Response: Thank you, this typo has been corrected on pg. 4 of the article document.

There is some repetition between the content of the "Sample size" paragraph and the "Primary and secondary outcome measures" paragraph regarding the MCID. As this concept is already well explained in the "Sample size" paragraph, I suggest that the authors delete the corresponding part in the "Primary and secondary outcome measures" one — specifically, the sentence starting with "A target effect size…".

Response: We agree and have removed this repetition from the outcome measure section.

Acronyms:
Could the authors please reduce the use of acronyms as much as possible? While all abbreviations may be familiar to the authors, they are not necessarily clear to readers, which sometimes makes the reading less comfortable. For example, HCPC is never defined, and PIS, PI, etc., could be clarified. It is often recommended to avoid acronyms in the abstract; they should be used only when absolutely necessary.

Response: We have carefully reviewed the article and can confirm that no acronyms have been used in the abstract without the full definition being given beforehand. Acronyms have been used to reduce word count wherever possible. However, we have added the full definition at other points in the document where this was not the case and thank the reviewer for the suggestion here.

Reviewer: 2
Dr. Brian McGuire, National University of Ireland, Galway,
Ireland Comments to the Author:
I am of the view that the authors have done an excellent job in addressing my suggestions and requests for clarification.

Response: We thank the reviewer for his comments.

Reviewer: 1
If you have selected 'Yes' above, please provide details of any competing interests.: 'Not applicable'

Reviewer: 2

If you have selected 'Yes' above, please provide details of any competing interests.: N/A
